# Account of Diseases in an Eastern District of London, from the 20th of Sept. to the 20th of October, 1807

**Published:** 1807-11

**Authors:** 


### *Account of Diseases in an Eastern District of London, from the 20th of Sept. to the 20th of October,* 1807.

| | |
|---|---|
| **ACUTE DISEASES.** | |
| Cholera - - - | 7 |
| Scarlatina Anginosa - | 6 |
| Icterus - - - | 2 |
| Synochus - - | 5 |
| Rubeola - - - | 2 |
| Rheumatismus Acutus | 3 |
| **CHRONIC DISEASES.** | |
| Tussis - - - | 15 |
| Dyspnœa - - - | 7 |
| Tussis cum Dyspnœa - | 15 |
| Hydrothorax - - | 1 |
| Phthisis Pulmonalis - | 3 |
| Hæmoptysis - - | 2 |
| Gastrodynia - - | 6 |
| Dyspepsia - - | 4 |
| Enterodynia - - | 3 |
| Diarrhœa - - | 4 |
| Colica Pictonum - | 2 |
| Menorrhagia - - | 5 |
| Fluor Albus - - | 7 |
| Dysuria - - - | 3 |
| Amenorrhœa - - | 2 |
| Rheumatismus Chronicus | 17 |
| **PUERPERAL DISEASES.** | |
| Ephemera - - | 7 |
| Menorrhagia Lochialis | 5 |
| Peritonitis - - | 1 |
| **INFANTILE DISEASES.** | |
| Aphthæ - - - | 2 |
| Ophthalmia Purulenta | 3 |
| Tabes Mesenterica - | 2 |
| Diarrhœa - - - | 6 |

The sudden change in the temperature of the air, which took place a few weeks ago, and the prevalence of the N. and N. E. winds, produced a very early return of those affections of the chest, which seldom appear to any great degree till the close of the autumn and commencement of the winter. These complaints, however, were much diminished in number, and in the violence of their symptoms, upon the return of a milder state of atmosphere. Scarlatina tina anginosa has occurred very frequently within the few past weeks. In this disease, after the symptoms which usually introduce every species of fever, an uneasiness is felt in the throat, together with a difficulty of deglutition; and this difficulty serves to distinguish it from another species of cynanche of a more malignant kind. A redness and tumour appear in the fauces, and soon afterwards a scarlet eruption is discovered upon the skin, which extends itself pretty generally over the surface; and after continuing three or four days, goes off, leaving behind a meally desquamation: the fever abates, and for the most part the disease terminates favourably. In one of the instances, however, referred to in the list, the disease proved fatal. In a little time after the eruption has disappeared, and the disease has in a great measure subsided, it frequently happens that a general anasarca supervenes; but this, for the most part, disappears upon the judicious exhibition of cathartic and diuretic remedies.

